# Association between Serum Soluble CD154 Levels and Mortality in Patients with Malignant Middle Cerebral Artery Infarction

**DOI:** 10.3390/ijms160612147

**Published:** 2015-05-28

**Authors:** Leonardo Lorente, María M. Martín, Agustín F. González-Rivero, Luis Ramos, Mónica Argueso, Juan J. Cáceres, Jordi Solé-Violán, Alejandro Jiménez, Juan M. Borreguero-León

**Affiliations:** 1 Intensive Care Unit, Hospital Universitario de Canarias, La Laguna 38320, Spain; 2 Intensive Care Unit, Hospital Universitario Nuestra Señora de Candelaria, Santa Cruz de Tenerife 38010, Spain; E-Mail: mar.martinvelasco@gmail.com; 3 Laboratory Deparment, Hospital Universitario de Canarias, La Laguna 38320, Spain; E-Mails: tinofran@hotmail.com (A.F.G.-R.); jmborreguero@hotmail.com (J.M.B.-L.); 4 Intensive Care Unit, Hospital General La Palma, Breña Alta 38713, Spain; E-Mail: lramosgomez@gmail.com; 5 Intensive Care Unit, Hospital Clínico Universitario de Valencia, Valencia 46004, Spain; E-Mail: moni_begasa@hotmail.com; 6 Intensive Care Unit, Hospital Insular, Las Palmas de Gran Canaria 35016, Spain; E-Mail: juanjose.caceresagra@gobiernodecanarias.org; 7 Intensive Care Unit, Hospital Universitario Dr. Negrín, Las Palmas de Gran Canaria 35010, Spain; E-Mail: jsolvio@gobiernodecanarias.org; 8 Research Unit, Hospital Universitario de Canarias, La Laguna 38320, Spain; E-Mail: ajimenezsosa@gmail.com

**Keywords:** sCD154, cerebral infarction, patients, mortality

## Abstract

Background: CD154 and its soluble counterpart (sCD154) are proteins of the tumor necrosis factor (TNF) family and exhibit proinflamatory and procoagulant properties. Higher circulating sCD154 levels have been found in ischemic stroke patients than in controls. However, the association between circulating sCD154 levels and mortality in ischemic stroke patients has not been reported, and was the focus of this study. Methods: This was a multicenter, observational and prospective study carried out in six Spanish Intensive Care Units. We measured serum sCD154 from 50 patients with severe malignant middle cerebral artery infarction (MMCAI), defined as Glasgow Coma Scale (GCS) lower than 9, at the moment of the severe MMCAI diagnosis and from 50 healthy controls. The end-point of the study was 30-day mortality. Results: We found higher serum sCD154 levels in patients with severe MMCAI than in healthy controls (*p* < 0.001). We found higher serum sCD154 levels (*p* < 0.001) in non-surviving (*n* = 26) than in surviving MMCAI patients (*n* = 24). Multiple binomial logistic regression analysis showed that serum sCD154 levels >1.41 ng/mmL were associated with 30-day mortality (OR = 10.25; 95% CI = 2.34–44.95; *p* = 0.002). Conclusions: The new more important finding of our study was that serum sCD154 levels in MMCAI patients were associated with mortality.

## 1. Introduction

Ischemic stroke is an important cause of disability, mortality and resources consumption [1]. CD154 is a protein of the tumor necrosis factor (TNF) family, and is expressed by platelets, B cells, monocytic cells, natural killer cells, mast cells, and basophils. CD40 is also expressed on T cells, and predominantly on T cells that promote inflammation. Th40 cells are highly associated with autoimmune diseases including type 1 diabetes and multiple sclerosis, and produces TNF and interleukin (IL)-6 [2]. CD154 and its soluble counterpart (sCD154) are proteins that exhibit proinflammatory and procoagulant properties when binding to their cell surface receptor CD154 [3]. CD154 is a member of the TNF receptor family that is expressed on the surface of many cells, such as endothelial cells, smooth muscle cells, B cells, monocytes, and microglia.

Higher CD154 platelet expression in ischemic stroke patients than in controls [4,5], higher circulating sCD154 levels in ischemic stroke patients than in controls [6,7,8,9,10,11,12,13], and higher CD154 platelet expression in ischemic stroke patients with poor functional outcome [14,15] have been reported. However the association between circulating sCD154 levels and mortality in ischemic stroke patients has not been reported, and that was the objective of this study.

## 2. Methods

### 2.1. Design and Subjects

This is a multicenter, observational, prospective study carried out in six Intensive Care Units of Spain. The study was approved by the Institutional Review Board of the six participant hospitals: Hospital Universitario de Canarias (La Laguna, Santa Cruz de Tenerife, Spain), Hospital Universitario Nuestra Señora de Candelaria (Santa Cruz de Tenerife, Spain), Hospital General de La Palma (La Palma, Spain), Hospital Clínico Universitario de Valencia (Valencia, Spain), Hospital Insular (Las Palmas de Gran Canaria, Spain), and Hospital Universitario Dr. Negrín (Las Palmas de Gran Canaria, Spain). Written informed consent from the patients or from their legal guardians was obtained.

We included 50 patients with severe malignant middle cerebral artery infarction (MMCAI) and 50 healthy volunteer control subjects. Severity of MMCAI was classified according to Glasgow Coma Scale (GCS) [16], and we included patients with GCS ≤8. Exclusion criteria were: age less than 18 years, pregnancy, inflammatory or malignant disease.

### 2.2. Variables Recorded

The following variables were recorded for each patient: sex, fibrinolityc therapy, decompressive craniectomy, age, temperature, sodium, glycemia, leukocytes, pressure of arterial oxygen (PaO_2_), PaO_2_/pressure of arterial oxygen/fraction inspired oxygen (FI0_2_) ratio, bilirubin, creatinine, hemoglobin, GCS, lactic acid, platelets, international normalized ratio (INR), activated partial thromboplastin time (aPTT), fibrinogen, Acute Physiology and Chronic Health Evaluation II (APACHE II) score [17]. The end-point of the study was 30-days mortality.

### 2.3. Blood Sample Collection

We recollected blood samples from 50 patients with severe MMCAI at the moment of the diagnosis to measure serum sCD154 levels, serum TNF-alpha levels, and plasma tissue factor (TF) levels. In addition, there were recollected blood samples from 50 healthy controls to measure serum sCD154 levels. To avoid the possible dispersion of serum level results, all the samples were processed at the same time and in the same laboratory, at the end of the recruitment process.

### 2.4. Laboratory Determinations

Venous blood samples were collected in serum separator tubes (SST) for determination of serum sCD154 and TNF-alpha levels, and in citrate tubes to determine plasma TF levels. Blood samples were centrifuged within 30 min at 1000× *g* for 15 min. The serum and plasma were removed and frozen at −80 °C until measurement. The determination of serum sCD154 and TNF-alpha levels, and plasma TF levels were centralized in the Laboratory Department of the Hospital Universitario de Canarias (La Laguna, Santa Cruz de Tenerife, Spain).

Serum sCD154 levels were assayed by specific ELISA (Bender MedSystems GmbH, Vienna, Austria). The intra-assay and inter-assay coefficients of variation (CV) were 4% (*n* = 8) and 6.8% (*n* = 8) respectively; and detection limits for the assays was 0.06 ng/mL.

Serum TNF-alpha levels were measured by a solid-phase, chemiluminiscents immunometrics assays kit (Immulite^®^, Siemens Healthcare Diagnostics Products, Llanberis, United Kingdom). The intra-assay and inter-assay CV were <3.6% (*n* = 20) and <6.5% (*n* = 20) respectively; and detection limits for the assays was 1.7 pg/mL.

Plasma TF levels were assayed by specific ELISA (Imubind^®^ Tissue Factor ELISA, American Diagnostica, Inc., Stanford, CT, USA). The intra-assay and inter-assay CV were <7.2% (*n* = 20) and <8% (*n* = 20) respectively; and detection limits for the assays was 10 pg/mL.

### 2.5. Statistical Methods

Continuous variables are reported as medians and interquartile ranges. Categorical variables are reported as frequencies and percentages. Comparisons of continuous variables between groups were carried out using Wilcoxon-Mann-Whitney test. Comparisons between groups on categorical variables were carried out with chi-square test.

Multiple binomial logistic regression analysis was applied to determine the independent contribution of serum sCD154 levels on 30-day mortality, controlling for GCS and age. Odds Ratio and 95% confidence intervals were calculated as measurement of the clinical impact of the predictor variables.

Receiver operating characteristic (ROC) analysis was carried out to determine the goodness-of-fit of the of serum sCD154 levels to predict 30-day mortality. Kaplan-Meier analysis of survival at 30 days and comparisons by log-rank test were carried out using serum sCD154 levels lower/higher than 1.41 ng/mL as the independent variable and survival at 30 days as the dependent variable. The association between continuous variables was carried out using Spearman’s rank correlation coefficient. A *p* value of less than 0.05 was considered statistically significant. Statistical analyses were performed with SPSS 17.0 (SPSS Inc., Chicago, IL, USA) and NCSS 2000 (Kaysville, UT, USA) and LogXact 4.1 (Cytel Co., Cambridge, MA, USA).

## 3. Results

**Figure 1:**
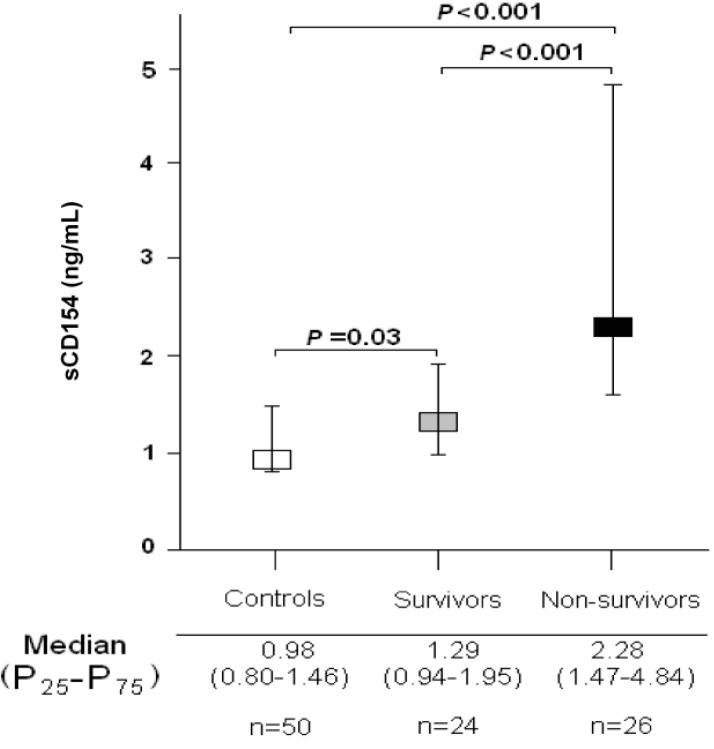
Serum sCD154 levels in severe malignant middle cerebral artery infarction patients and healthy controls.

Table 1 shows the comparisons of age, sex and serum sCD154 levels between patients with severe MMCAI patients (*n* = 50) and healthy controls (*n* = 50). There were no significant differences between patients with severe MMCAI and healthy controls groups on age and sex. However, we found higher serum sCD154 levels in patients with severe MMCAI patients than in healthy controls (*p* < 0.001); in addition, serum sCD154 levels were higher in surviving (*p* = 0.03) and non-surviving patients with severe MMCAI patients (*p* < 0.001) compared to healthy controls (Figure 1).

**Table 1:** Characteristics of healthy controls and patients with severe MMCAI.

	Healthy Controls (*n* = 50)	Patients (*n* = 50)	*p*-Value
Gender female—*n* (%)	15 (30.0%)	17 (34.0%)	0.83
Age—median years (p 25–75)	59 (50–68)	60 (51–69)	0.47
Serum sCD154—median ng/mL (p 25–75)	0.98 (0.80–1.46)	1.79 (1.22–3.56)	<0.001

Table 2 shows the comparisons between non-surviving (*n* = 26) and surviving (*n* = 24) MMCAI patients. There were no significant differences between non-surviving and surviving patients in temperature, sodium, PaO_2_, PaO_2_/FI0_2_ ratio, lymphocytes, leukocytes, lactic acid, INR, hemoglobin, glycemia, GCS score, sex, fibrinogen, creatinine, bilirubin, aPTT, APACHE-II score and age. We found that non-surviving showed compared to surviving patients lower platelet count, and higher circulating levels of TNF-alpha, TF, and sCD154 (*p* < 0.001). In addition, non-surviving patients had a lower ICU stay duration than surviving (4 (2–9) *vs*. 23 (16–40) days; *p* < 0.001).

**Table 2:** Clinical and biochemical characteristics of MMCAI patients according to 30-day survival.

	Survivors (*n* = 24)	Non-Survivors (*n* = 26)	*p* Value
TNF-alpha (pg/mL)—median (p 25–75)	9.25 (9.02–10.63)	12.95 (10.03–15.08)	0.01
TF (pg/mL)—median (p 25–75)	156 (127–196)	279 (181–400)	0.02
Temperature (°C)—median (p 25–75)	36.5 (35.7–37.0)	37.0 (35.7–37.8)	0.26
Sodium (mEq/L)—median (p 25–75)	140 (138–145)	140 (137–146)	0.91
sCD154 (ng/mL)—median (p 25–75)	1.29 (0.94–1.95)	2.28 (1.47–4.84)	<0.001
Platelets—median × 10^3^/mm^3^ (p 25–75)	227 (183–308)	152 (123–190)	0.003
PaO_2_ (mmHg)—median (p 25–75)	110 (101–194)	104 (85–139)	0.10
PaO_2_/FI0_2_ ratio—median (p 25–75)	246 (192–327)	248 (175–320)	0.41
Lymphocytes—median × 10^3^/mm^3^ (p 25–75)	1.5 (0.9–1.8)	1.1 (0.5–2.1)	0.30
Leukocytes—median × 10^3^/mm^3^ (p 25–75)	12.8 (9.8–16.9)	14.4 (11.9–21.9)	0.49
Lactic acid (mmol/L)—median (p 25–75)	1.25 (0.93–1.68)	1.50 (1.01–3.15)	0.08
INR—median (p 25–75)	1.07 (1.01–1.20)	1.20 (1.07–1.48)	0.16
Hemoglobin (g/dL)—median (p 25–75)	12.0 (11.3–13.8)	12.0 (11.0–15.1)	0.92
Glycemia (g/dL)—median (p 25–75)	133 (105–170)	135 (110–154)	0.92
GCS score—median (p 25–75)	7 (6–8)	6 (4–8)	0.10
Gender female—*n* (%)	8 (33.3)	9 (34.6)	0.99
Fibrinogen (mg/dL)—median (p 25–75)	440 (335–494)	409 (322–598)	0.71
Decompressive craniectomy—*n* (%)	7 (29.2)	5 (19.2)	0.51
Creatinine (mg/dL)—median (p 25–75)	0.80 (0.60–1.10)	1.01 (0.85–1.45)	0.052
Bilirubin (mg/dL)—median (p 25–75)	0.50 (0.38–0.90)	0.53 (0.30–1.20)	0.76
aPTT (seconds)—median (p 25–75)	28 (25–29)	26 (25–33)	0.96
APACHE-II score—median (p 25–75)	20 (16–25)	22 (19–29)	0.14
Age (years)—median (p 25–75)	47 (32–67)	66 (45–76)	0.14

p 25–75 = percentile 25th–75th; PaO_2_ = pressure of arterial oxygen/fraction inspired oxygen; FIO_2_ = pressure of arterial oxygen/fraction inspired oxygen; TNF = tumor necrosis factor; TF = tissue factor; INR = international normalized ratio; GCS = Glasgow Coma Scale; aPTT = activated partial thromboplastin time; APACHE II = Acute Physiology and Chronic Health Evaluation.

We found in multiple binomial logistic regression analysis that serum sCD154 levels >1.41 ng/mmL were associated with 30-day mortality (OR = 10.25; 95% CI = 2.34–44.95; *p* = 0.002) controlling for GCS and age (Table 3).

**Table 3:** Multiple binomial logistic regression analysis to predict 30-day mortality.

Variable	Odds Ratio	95% Confidence Interval	*p*
Serum sCD154 > 1.41 ng/mmL	10.25	2.34–44.95	0.002
GCS score	0.72	0.49–1.06	0.09
Age (years)	1.05	0.99–1.11	0.08

**Figure 2:**
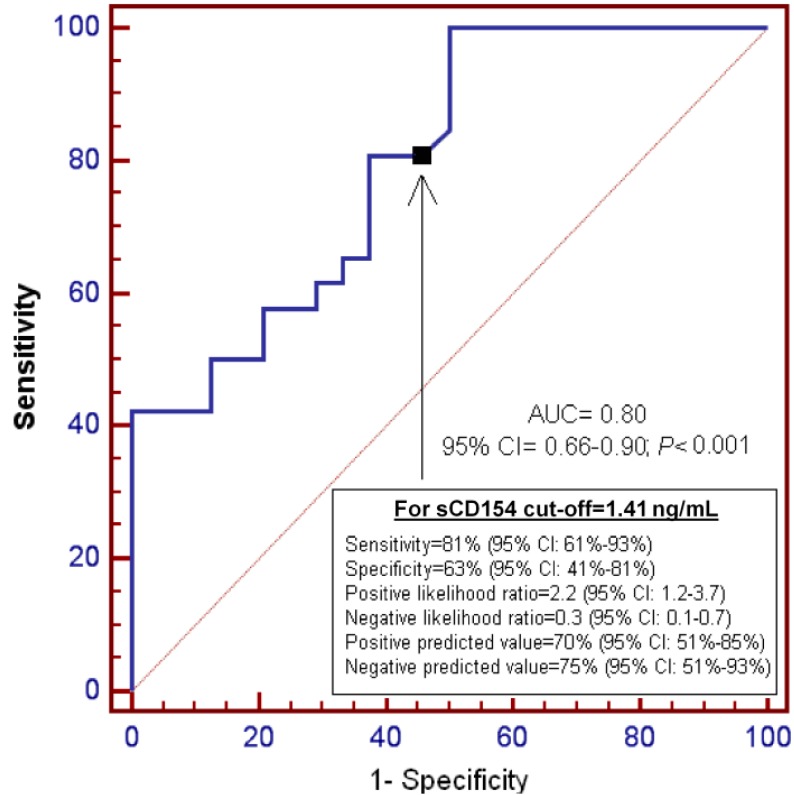
Receiver operation characteristic analysis using serum sCD154 levels as predictor of mortality at 30 days.

Figure 2 shows ROC analysis of serum sCD154 levels to predict 30-day mortality. We found that the area under the curve (AUC) was 0.80 (95% CI = 0.66–0.90; *p* < 0.001). Diagnostic goodness-of-fit for serum CD154 levels of 1.41 ng/mL were the following: sensitivity = 81% (95% CI = 61%–93%), specificity = 63% (95% CI = 41%–81%).

**Figure 3:**
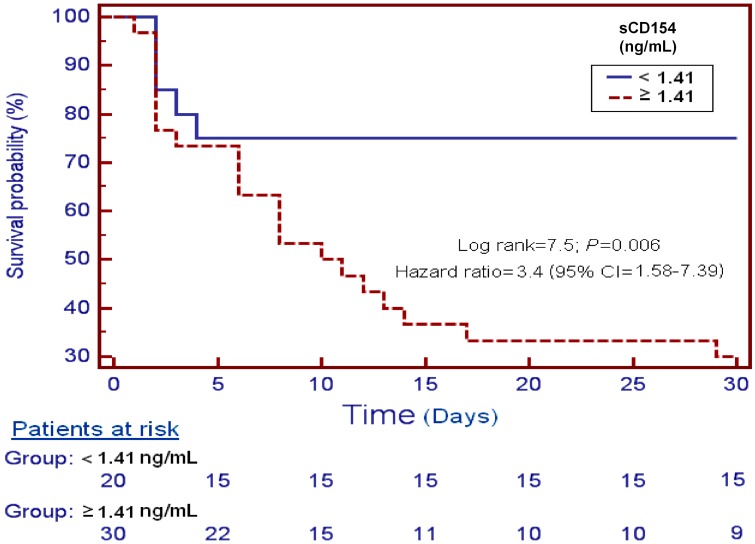
Survival curves at 30 days using serum sCD154 levels higher or lower than 1.41 ng/mL.

Figure 3 shows survival analysis of patients with serum sCD154 levels higher and lower of 1.41 ng/mmL, and survival at 30 days as the dependent variable. We found that patients with serum sCD154 levels higher than 1.41 ng/mmL showed higher mortality at 30 days than patients with lower levels (Hazard ratio = 3.4; 95% CI = 1.58–7.39; *p* = 0.006).

We found an association of circulating levels of sCD154 with GCS (rho = −0.21; *p* = 0.04), TF (rho = 0.52; *p* < 0.001) and TNF-alpha (rho = 0.54; *p* < 0.001).

## 4. Discussion

The novel findings of our study were the following: (a) non-surviving severe MMCAI patients had higher serum sCD154 levels than surviving patients; (b) there is an association between circulating levels of sCD154, GCS, TF and TNF-alpha in patients with severe MMCAI; (c) serum sCD154 levels could be used as prognostic biomarker in patients with severe MMCAI.

We found higher serum sCD154 in MMCAI patients than in controls, and previously, higher CD154 platelet expression in ischemic stroke patients than in controls were reported [4,5], as well as higher circulating sCD154 levels in ischemic stroke patients than in controls [6,7,8,9,10,11,12,13].

Previously higher CD154 platelet expression in ischemic stroke patients with poor functional outcome [14,15] was reported. However, in our study we found for the first time, an association between circulating levels of sCD154 and stroke severity assessed by GCS, and higher serum sCD154 levels in non-surviving severe MMCAI patients than in surviving patients, an association between serum sCD154 levels and mortality in logistic regression analysis, and that serum sCD154 levels could predict mortality according to the ROC analysis. The findings are in consonance with the results of previous studies showing higher sCD154 in ischemic stroke patients with poor functional outcome [14,15] and with the findings of other studies reporting an association between serum sCD154 levels and mortality in patient with acute coronary syndrome [18], sepsis [19,20] and brain trauma injury [21].

Circulating sCD154 levels in MMCAI patients could play a physiological role by its proinflammatory [22,23] and procoagulant [24,25,26,27,28,29] actions. CD154 is stored in α-granules of unstimulated platelets and when platelets become activated, CD154 translocates to the platelet surface. Then CD154 is cleaved by MMP-9 and later released into blood circulation as sCD154 [30]. Afterwards, sCD154 binds to its receptor CD40 in different cells, such as endothelial cells mononuclear phagocytes triggers the expression of various proinflammatory mediators, such as the interleukin (IL)-1, IL-6, IL-12, TNF-alpha, and interferon-gamma [23]. In addition, sCD154 could have prothrombotic effects via induction of TF [24,25,26,27] and binding to the glycoprotein IIb/IIIa platelet receptor [28,29]. Interestingly, we report for the first time a positive association of circulating levels of sCD154 with TF and TNF-alpha in patients with severe MMCAI patients. The association between serum levels of sCD154 and TNF-alpha, a pro-inflammatory cytokine, could involve a higher inflammatory state in those patients with higher serum sCD154 levels and higher risk of death. The association between serum sCD154 and plasma TF levels, a prothrombotic factor, could facilitate that patients with higher circulating sCD154 levels are associated with the development of vascular thrombosis, brain ischemia and death of the patient.

Another interesting findings of our study were that we found lower platelet count in non-surviving MMCAI patients than in surviving patients. These findings are in agreement with those of the study by D’Erasmo *et al.* [31]. In that study, the authors found an association between early platelet count reduction and infarct extension and clinical outcome in patients with ischemic cerebral infarction; and they believed that the results demonstrate that the platelet consumption and/or accumulation in the infarct area, expressed by circulating platelet decrease, is related to the severity of neurological involvement, infarct size and poor clinical outcome.

The administration of modulators of sCD154 activity could have a potential beneficial effect in MMCAI patients. In several studies the use of statins has been found to decrease circulating sCD154 levels in patients with coronary artery disease [32,33,34]. In addition, in a meta-analysis the use of statin therapy at stroke onset was associated with improved outcome and reduced fatality [35].

Some limitations of our study should be recognized. First, we did not report data of circulating sCD154 during follow-up. Second, the determination of other inflammatory cytokines and coagulation biomarker could be interesting. Third, we found that serum sCD154 levels were associated with mortality controlling for GCS score and age; however other factors could have played a role in mortality, and thus, larger series of patients including more variables in a single multiple logistic regression analysis are needed to confirm our findings.

We believe, that according to the results of our study, the determination of serum sCD154 levels at the moment of the MMCAI diagnosis could be used to prediction the outcome and could generate interest for research into the use of modulator agents of sCD154 activity in these patients.

## 5. Conclusions

The novel and most important finding of our study is that serum sCD154 levels in MMCAI patients are associated with mortality.
